# Synthesis of TiO_2_ Nanobelt Bundles Decorated with TiO_2_ Nanoparticles and Aggregates and Their Use as Anode Materials for Lithium-Ion Batteries

**DOI:** 10.3390/mi14020243

**Published:** 2023-01-18

**Authors:** Wenpo Luo, Juliette Blanchard, Domenica Tonelli, Abdelhafed Taleb

**Affiliations:** 1 PSL Research University, Chimie ParisTech—CNRS, Institut de Recherche de Chimie Paris, 75231 Paris, France; 2 Laboratoire de Réactivité de Surface (LRS), Sorbonne Université, CNRS, UMR 7197, 4 Place Jussieu, 75005 Paris, France; 3 Department of Industrial Chemistry “Toso Montanari”, University of Bologna, 40136 Bologna, Italy; 4 Sorbonne Université, 4 place Jussieu, 75231 Paris, France

**Keywords:** mesoporous TiO_2_, pores architecture, specific surface area, lithium-ion batteries

## Abstract

TiO_2_ nanobelt bundles decorated with TiO_2_ aggregates were prepared using an easy and scalable hydrothermal method at various temperatures (170, 190, 210, and 230 °C). It was demonstrated that the synthesis temperature is a key parameter to tune the number of aggregates on the nanobelt surface. Prepared TiO_2_ aggregates and nanobelt bundles were used to design anode materials in which the aggregates regulated the pore size and connectivity of the interconnected nanobelt bundle structure. A galvanostatic technique was employed for the electrochemical characterization of TiO_2_ samples. Using TiO_2_ as a model material due to its small volume change during the cycling of lithium-ion batteries (LIBs), the relationship between the morphology of the anode materials and the capacity retention of the LIBs on cycling is discussed. It was clearly found that the size and connectivity of the pores and the specific surface area had a striking impact on the Li insertion behavior, lithium storage capability, and cycling performance of the batteries. The initial irreversible capacity was shown to increase as the specific surface area increased. As the pore size increased, the ability of the mesoporous anatase to release strain was stronger, resulting in better cycling stability. The TiO_2_ powder prepared at a temperature of 230 °C displayed the highest discharge and charge capacities (203.3 mAh/g and 140.8 mAh/g) and good cycling stability.

## 1. Introduction

Global warming, which threats our ecosystem’s equilibrium, is becoming a great concern. Furthermore, it is well-accepted that fossil energy contributes strongly to global warming and that renewable energy sources could be serious candidates to replace fossil energy. The issue of how to store renewable energy and use it when needed has driven the industrial and scientific communities to develop energy storage devices. Batteries [1,2] and supercapacitors [3,4] have attracted a lot of attention due to their portability, low cost, and energy storage effectiveness. Furthermore, another motivation for battery development is the emergence of electric vehicles [5,6] as potential candidates to replace fuel engine ones, which consume fossil energy and are a major source of pollution.

Lithium-ion batteries (LIBs) are currently one of the most popular energy storage systems for portable electronic devices [5,7,8,9,10,11]. This is mainly due to their high energy density, high life cycle, and safety. Lithium-ion batteries are composed of positive and negative electrodes separated by an electrolyte and are usually made of polypropylene–polyethylene and dissociated salts of Li in alkyl organic carbonates. The two electrodes are isolated by a separator, which allows Li diffusion during the charging and discharging processes. For the cathode, which is the positive electrode, the commonly used materials are based on transition metals oxides or phosphates, such as LiMnO_2_ [12], LiCoO_2_ [13], LiNiO_2_ [14], LiFePO_4_ [15], LiMnPO_4_ [16], etc. For the negative electrode (anode), graphite is the commonly used active material because of its abundance, low cost, and excellent cyclability at high charge rates (C-rates). However, its main drawbacks are low power density; the poor stability of its layered structure, which collapses and exfoliates on cycling; low capacity; secondary reactions with the electrolyte to form a solid electrolyte interface (SEI); and safety concerns [17,18,19,20,21].

However, there is an urgent need for the development of alternative anode materials with high capacities and high lithium-ion diffusion rates that could improve the energy and power densities of LIBs. Titanium dioxide (TiO_2_) has been successfully demonstrated to be a promising material for energy storage applications [10,22,23,24] due to its excellent electrochemical performance and other properties, such as non-hazardous handling, low cost, low toxicity, good cycling life, appropriate insertion potential (∼2.0 V), and low volume expansion (3–4%) during lithium insertion [25,26,27]. TiO_2_ exists in different phases: rutile [28], anatase [29], and brookite [30]. The anatase phase is the most studied host material for electroactive Li insertion [31,32,33]. However, due to their low ionic diffusion and electronic conductivity, large TiO_2_ anatase particles demonstrate unsatisfactory performance, which hinders their practical application. To improve the performance of TiO_2_ anatase particles, different approaches have been adopted, including tuning their crystallinity, size, morphology, and specific surface area [34,35,36,37]. TiO_2_ nanomaterials with large specific surface areas allow for the enhancement of lithium storage capability and cyclability rate due to a short diffusion pathway [31,32,33,34]. Furthermore, Li-ion diffusion is also controlled by the pore length because of particle size and morphology [38]. It has been reported that the pore length has a strong influence on the capacity and the cyclability rate of mesoporous Co_3_O_4_ as a cathode material for LIBs, which increase when the nanoparticle size decreases [38]. It is well accepted that with mesoporous material, the high specific surface area favors Li ion diffusion, which leads to a high specific capacity and cyclability rate. At the same time, controlling the architecture of materials for rechargeable batteries is one of the main challenges in the design and synthesis of anode materials. The correlation between the architecture of anode materials and the performance of rechargeable batteries plays an important role in guiding the controlled preparation of the materials. Various works have reported the existence of a relationship between the morphology of the materials and the performance of LIBs, which is not evident from the breaking down of these materials during battery cycling and the evolution of their morphology.

In this study, TiO_2_ nanobelt bundles decorated with TiO_2_ aggregates were successfully prepared by a hydrothermal procedure. The synthesis temperature was pointed out to be a crucial parameter to tune the TiO_2_ powder design. Using TiO_2_ as a model material due to its small volume change during LIB cycling, the relationship between the morphology of anode materials and the capacity retention of LIBs on cycling is discussed, emphasizing the role of the specific surface area and pore properties in terms of size and connectivity.

## 2. Materials and Methods

### 2.1. Synthesis of TiO_2_ Nanoparticles

**Scheme 1:**
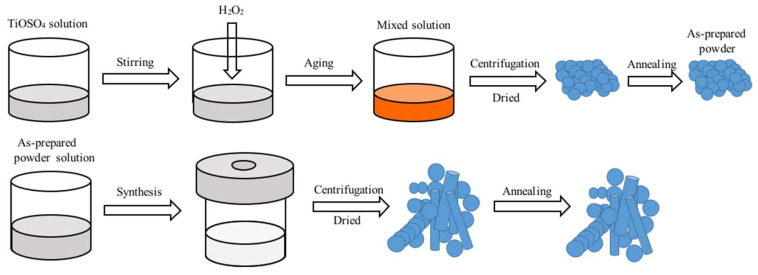
Schematic illustration of the different steps for TiO_2_ powder synthesis.

All the chemical reagents were purchased from Sigma Aldrich Chemical Reagent Co. Ltd. and were used without further purification unless otherwise stated. The water used in all the experiments was purified by a Milli Q System (Millipore, electric resistivity 18.2 MΩ·cm). The TiO_2_ powders were prepared according to Scheme 1 using a hydrothermal synthesis method.

To prepare the precursor solution, 0.64 g of TiOSO_4_ and 16 mL of deionized water were mixed, followed by the addition of 1.266 g of H_2_O_2_ under stirring at 25 °C. After 24 h of aging at room temperature, the solution became orange, and the precipitate was collected by centrifugation, washed with water six times and then with ethanol twice, and finally dried overnight in an oven at 50 °C. The obtained powder was subjected to thermal annealing at a temperature of 500 °C for 1 h. A suspension was prepared by dispersing 0.2 g of the prepared TiO_2_ powder in 16 mL of cold water under an ultrasonic bath for 15 min. Then the solution was placed in a PTFE-lined autoclave (volume: 25 mL) and heated at a rate of 2.5 °C/min. Four temperatures were selected for the syntheses: 170, 190, 210, and 230 °C, which were maintained for 16 h. Then, the synthesized TiO_2_ powders were recovered by centrifugation, washed with water six times and with ethanol twice, and dried overnight in the oven. Finally, the dried powders were annealed in air at 500 °C for 1 h with a heating rate of 5 °C/min.

### 2.2. Characterization of TiO_2_ Films

The morphologies of the prepared powders were investigated with a high-resolution Ultra 55 Zeiss FEG scanning electron microscope (FEG-SEM) operating at an acceleration voltage of 10 kV. The crystalline structure of the TiO_2_ powders was confirmed by an X-ray diffractometer (Siemens D5000 XRD unit) in a 2θ range from 20° to 80° by 0.07° s^−1^ increasing steps, operating at a 40 kV accelerating voltage and a 40 mA current and using a Cu Kα radiation source with λ = 1.5406 Å.

Nitrogen adsorption-desorption isotherms were obtained at the liquid nitrogen temperature with a Belsorp Max apparatus. Before analysis, the samples were degassed at 200 °C for 3 h. The specific surface area was evaluated using the Brunauer–Emmett–Teller (BET) method in the P/P° range of 0.05–0.25. The pores size distribution was determined from the adsorption branch of the isotherm using the Grand Canonical Monte Carlo (GCMC) method. The total pore volume was determined from the amount of N_2_ adsorbed up to P/P° = 0.98.

### 2.3. Electrochemical Measurements

Electrodes were fabricated by intimately mixing the active material (80 wt%) with mesoporous carbon (~7 wt%), graphite (~7 wt%), and binder PTFE (~7 wt%) as a binder. The resulting mixture was then compressed onto a stainless steel foil and allowed to dry overnight at 80 °C. Swagelok-type electrochemical cells were assembled in a glove box under a dry argon atmosphere using lithium metal as a counter electrode and a Celgard 2400 membrane with glass fiber as a separator. Cells were kept in a glove box for 12 h before electrochemical measurements. The electrolyte was prepared by dissolving 1 M LiPF_6_ in a 1:1:1 volume ratio of ethylene carbonate (EC), diethyl carbonate (DEC), and dimethyl carbonate (DMC). All cells were tested at room temperature in the range of 1–3.0 V vs. Li^+^/Li using a program-controlled battery test system (EC-Lab, France) at a current rate of 0.12 C.

## 3. Result and Discussion

### 3.1. Phase and Microstructure Characterization

**Figure 1:**
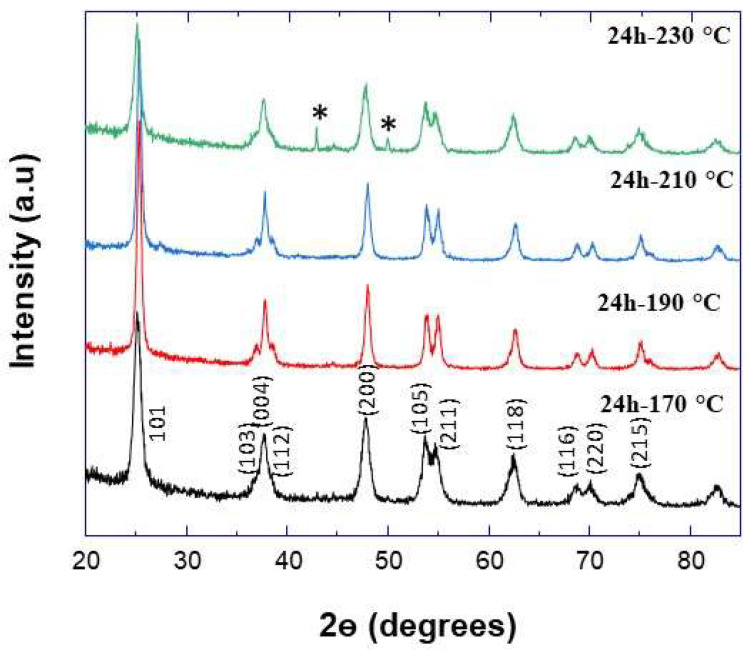
XRD pattern of TiO_2_ nanostructures prepared at the different temperatures indicated. The peaks highlighted with stars correspond to those of the substrate.

The crystallinity of the powders obtained at various temperatures was investigated by XRD, and the obtained patterns are depicted in Figure 1. The powders were crystalline, and all the reflections were assigned to the TiO_2_ anatase phase (JCPDS No. 89-4921), with no other detected phases. The average TiO_2_ crystallite sizes were calculated with the Scherrer formula, (D = 0.9 λ/Bcosθ), using the full width at half the maximum of the peak intensity corresponding to the (101) crystallographic plane [39]. The crystallite sizes of TiO_2_ powders are given in Table 1. It is clear that the size of the crystalline domain decreases as the synthesis temperature increases.

**Figure 2:**
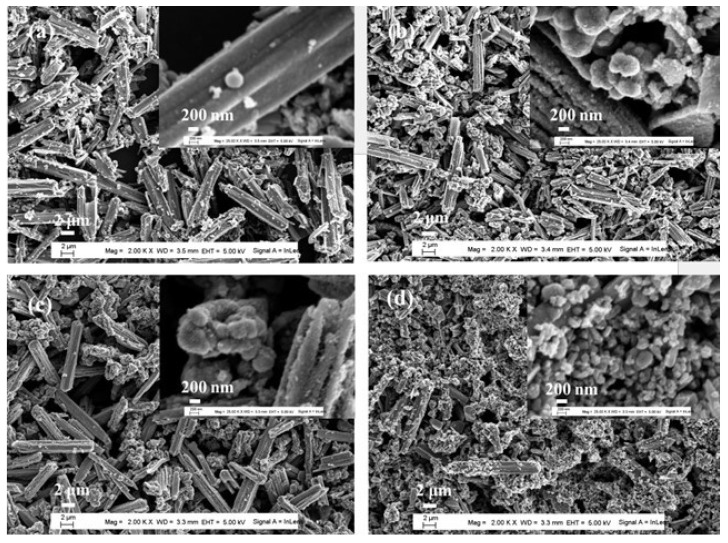
FEG-SEM images of prepared TiO_2_ powders at various synthesis temperatures: (**a**) 170 °C; (**b**) 190 °C; (**c**) 210 °C; and (**d**) 230 °C, inserts are the corresponding high magnification.

The morphology of the TiO_2_ powders was investigated using FEG-SEM (Figure 2). It is worth noting that the bundles consisted of the assembly of nanobelts. For all samples, both nanobelt bundles and aggregates resulting from the spontaneous aggregation of TiO_2_ nanoparticles and nanobelts were observed. In the regions indicated in the insert of Figure 2b, it is possible to observe the presence of a curved nanobelt with a thickness of ~200 nm, which was less than its width of about 600 nm. On the other hand, in the insert of Figure 2a,c and the other regions of Figure 2a–c, it is possible to observe a stack of several nanobelts with steps of about 200 nm corresponding to their thickness. It can be noticed that the widths of the nanobelts varied from 400 nm to 2 µm, and their lengths varied from 4 µm to 15 µm. It was also shown that the aggregates ranged in size from 100 to 600 nm (Figure 2) and were made of nanoparticles. In previous studies, it has been shown that these aggregates are porous assemblies of nanoparticles [40,41]. Furthermore, both the number of nanoparticles and their aggregation increased with the synthesis temperature.

**Figure 3:**
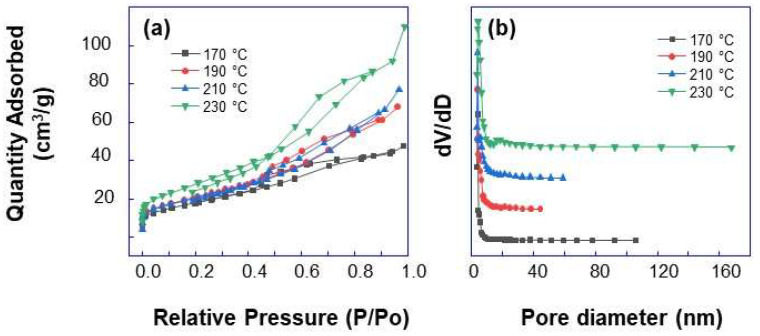
(**a**) N_2_ adsorption–desorption isotherms and (**b**) GCMC pore size distribution curves for the TiO_2_ powders prepared at various synthesis temperatures.

The adsorption–desorption isotherms are shown in Figure 3a, and the pore size distribution (obtained from the isotherm adsorption branches using the GCMC model) is shown in Figure 3b. Nitrogen adsorption/desorption isotherms of all the prepared TiO_2_ powders showed a hysteresis loop, indicating that they exhibited high mesoporosity [42] associated with relatively large pores ranging from 4.0 to 4.5 nm. The pronounced hysteresis is believed to be connected to capillary condensation at large pore channels, which can be also associated with the modulation of the channel structure [43]. The obtained results for the TiO_2_ powders in terms of specific surface area, average pore diameter, and pore volume are summarized in Table 2. The specific surface area and, above all, the pore volume constantly increased with the increase in the synthesis temperature, which was consistent with the transition from powders, in which nanobelt bundles were dominant to powders where the bundles were covered by a high number of nanoparticles and aggregates (Figure 2). Therefore, the increasing synthesis temperature promoted nanoparticle formation and aggregation at the surface of the nanobelt bundles. In addition, the porosity of the nanobelt bundles changed as a function of the temperature, and the phenomenon of nanoparticle formation and aggregation could also enlarge the pore size among the nanobelt bundles. It is worth mentioning that the prepared TiO_2_ structures enable a rapid filling of mesopores by the electrolyte during the electrochemical charging/discharging cycles, which enhances the rate capability of the anode materials [33].

### 3.2. Electrochemical Performance

The powders prepared at various temperatures were tested as anode materials for LIBs. The insertion of Li^+^ into anatase powders is well accepted to be accompanied by a phase transition from tetragonal TiO_2_ (space group I41/amd) to orthorhombic Li_0.5_TiO_2_ (space group Imma), according to the reaction:

TiO_2_ + xLi^+^ + xe^−^ → Li_x_TiO_2_ (1)

where x is the amount of inserted Li^+^ in the anatase, which depends both on the crystallography and the microstructure of the materials [44]. Although anatase possesses a theoretical specific capacity of 335 mAh/g, only half of this capacity is usually observed. This is mainly due to the strong Li-Li repulsions in the Li_x_TiO_2_ lattice at insertion ratios greater than 0.5 [45,46]. The main redox reaction underlying the TiO_2_ electrochemical activity is that corresponding to the interconversion of Ti^4+^/Ti^3+^ during discharging/charging cycles.

**Figure 4:**
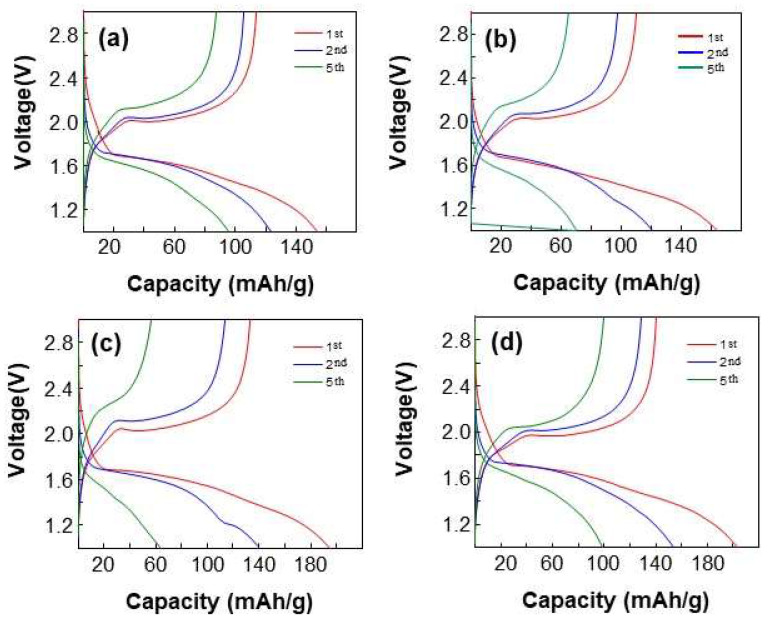
Charging/discharging curves of TiO_2_ powders prepared at the various synthesis temperatures:(**a**) 170 °C; (**b**) 190 °C; (**c**) 210 °C; and (**d**) 230 °C cycled between 1.0 and 3.0 V at a constant current rate of 0.12 C.

In Figure 4, the first, second, and fifth charging/discharging cycles relevant to the TiO_2_ powders synthesized at 170, 190, 210, and 230 °C are displayed. Electrochemical cycling was performed at voltages ranging from 1.0 to 3.0 V and at a constant current rate of 0.12 C. The curves show similar patterns with clear plateaus near ~1.75 V for both discharging and charging cycles and display values that are similar to others reported for the same systems [47,48]. It is worth noting that the capacity increased with the synthesis temperature, and the highest discharging and charging capacities (203.3 mAh/g and 140.8 mAh/g, respectively) were obtained for the powder synthesized at the highest temperature, 230 °C. The observed irreversible capacity during the first cycle was mainly attributed to three phenomena: the formation of a solid solution [49,50], the irreversible lithium insertion in the crystal lattice defects or on the electrode surface sites [51], and the reduction of the electrolyte on the electrode surface, leading to the formation of a passivating layer named the solid electrolyte interphase layer (SEI) [52]. The highest capacity observed for the electrode fabricated with TiO_2_ powder synthesized at 230 °C was explained by the higher specific surface area and larger pore size, which offered a high contact area between the active material and the electrolyte.

**Figure 5:**
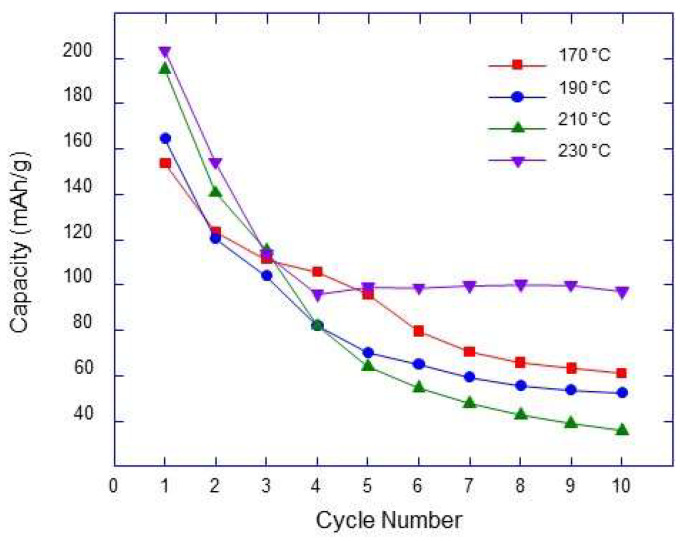
Cycling performance at a constant current rate (0.12 C) of TiO_2_ powders prepared at the reported temperatures and employed as anode materials for LIBs.

The cycling performances (up to 10 cycles) of four LIBs using TiO_2_ powders synthesized at different temperatures as anode material are presented in Figure 5. As can be seen, two behaviors can be distinguished. A rapid decrease in capacity after the first few cycles (~ 4 or 5) is evident, followed by a much slower trend. The rapid degradation of capacity during the first cycles is partly due to the formation of the SEI passivating layer because of the decomposition of the electrolyte, along with the irreversible reaction between the Li ions and the TiO_2_ materials of the anode, which consumes a large amount of Li^+^. Another reason is that the TiO_2_ powder underwent a change in volume during the insertion/de-insertion cycles of the Li ions, causing a non-uniform distribution of internal stress, which induced the breaking down of the initial particles into small particles, with a probable loss of electrical contact with the rest of the active anode material. The TiO_2_ powder synthesized at 230 °C showed the best cycling performance with a very slow decrease in capacity after the fifth cycle. The surface of the nanobelt bundles of this TiO_2_ powder was characterized by high coverage of TiO_2_ aggregates, which were responsible not only for the increased specific surface area but also for the larger pore size and, more importantly, pore volume. Furthermore, as the TiO_2_ synthesis temperature increased, the initial capacity also increased, and this was consistent with the higher specific surface. The trend does not explain the behavior of the capacity after the fifth cycle for the powders prepared at 170, 190, and 210 °C, which displayed a decrease in capacity, even if slight, with the number of cycles. Furthermore, the data obtained for TiO_2_ powders clearly showed that as the pore size increased, the specific surface area also increased. These results disagree with the classical model of spherical nanoparticles, in which the surface area corresponds to the geometric surface of the particles, and thus increases as the particle size decreases, while the interstitial pores, created by the packing of the spherical nanoparticles, decrease. To explain this anomaly, it is crucial to assume that nanobelt bundles play a role in fixing pore size and volume and that aggregates on their surface increase the pore size among the nanobelt bundles and change their connectivity. Furthermore, it is well accepted that the internal structure of mesoporous materials accommodates the volume expansion during the lithium insertion and releases the deformation stress [53]. From the results for the TiO_2_ powder synthesized at 230 °C, it should be noted that the pore volume was significantly higher than the one displayed by the other three TiO_2_ powders, and that means a more flexible pores connectivity with a better capability of releasing deformation stress. This TiO_2_ powder with micro- and meso-porous structures better accommodated the volume change during the discharging/charging cycles, which explains the observed roughly constant capacity after the fourth cycle (Figure 5), resulting in a much better cycling performance than the other three samples. With the latter TiO_2_ powders, the increase in the synthesis temperature induced the increase of nanoparticles and their aggregation covering the surface of the nanobelt bundles, thus leading to a higher specific surface and a reduction in the pore length; these properties favored the increase in the initial capacity (first cycle). Unfortunately, the new connectivity and pore structure were less flexible and were not able to accommodate the deformation stress due to the change in volume of the anode during the discharge/charge cycles, resulting in the formation of electrically isolated grains. This effect would greatly reduce the capacity of the batteries if submitted to the discharge/charge cycles. This effect was enhanced by temperature, as can be observed for temperatures of 170 °C, 190 °C, and 210 °C, suggesting that the porous structures favored at intermediate synthesis temperatures have a reduced ability to accommodate volume change during discharge/charge cycles.

**Figure 6:**
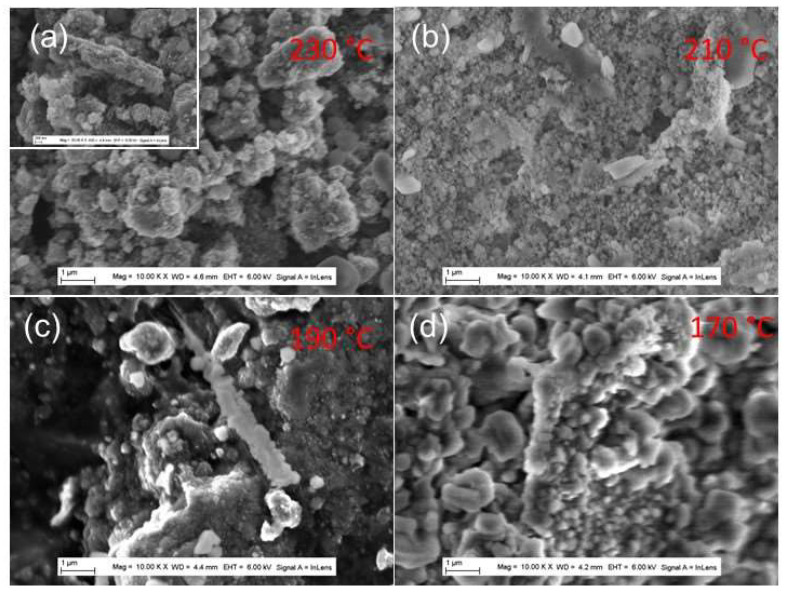
FEG-SEM pictures after the 10th cycle at constant current rate (0.12 C) of the TiO_2_ powders prepared at the indicated temperatures and used as anode materials for LIBs, the (a) insert is the corresponding high magnification.

To check whether other parameters influence the observed behavior, the powders were characterized by XRD and FEG-SEM after the 10th cycle. It can be observed from the FEG-SEM characterization (Figure 6) that only aggregates could be observed, whereas nanobelt bundles seemed to be completely absent. This supports the breakup of the nanobelt bundles during the cycling processes and their transformation into aggregates. The change in morphology of the TiO_2_ powders synthesized at 170, 190, 210 °C, and 230 °C was due to the volume variation upon Li^+^ intercalation and de-intercalation, which induces stress within the materials. Another important observation is the size of the aggregates, which was different for the powders synthesized at various temperatures and controls the interconnectivity of the pores together with the size of the TiO_2_ nanoparticles. This could explain the slow decrease in the specific capacity of lithium-ion batteries after the fifth cycle. The significant size change of the samples during the charge–discharge cycles was also confirmed by the XRD experiments, as shown in Table 3. The notable decrease in the TiO_2_ crystallite size compared to their initial size is evident after the 10th cycle, which supports the crystalline breakdown occurring during the cycling processes. Considering the results of XRD and the capacity as a function of the number of cycles, it can be concluded that the aggregates formed by small particles better buffer the volume variation during the battery cycling, which may explain the better capacity of the powder prepared at 230 °C. After the fifth cycle, the capacity of the battery was roughly constant, and it was controlled by the properties of the nanoparticle aggregates resulting from the nanobelt bundles’ disintegration during the first cycles. A previous study reported that the specific surface area and the pore size strongly influenced the Li-ion batteries’ performance [41].

## 4. Conclusions

TiO_2_ nanobelt bundles decorated with TiO_2_ aggregates were successfully prepared using an easy and scalable hydrothermal method at various temperatures of 170, 190, 210, and 230 °C. It was proven that temperature is a crucial parameter to control the properties of TiO2 powders in terms of the specific surface area, pore size, and pore volume. In particular, when the synthesis temperature increased, the morphology of TiO_2_ powders changed from nanobelt bundles with low to high coverage of nanoparticles and aggregates on their surface. The electrochemical measurements showed that the TiO_2_ powder synthesized at a temperature of 230 °C exhibited the highest discharging and charging capacities (203.3 mAh/g and 140.8 mAh/g considering the first cycle) and the best cycling performance. This was explained by the higher specific surface area of the TiO_2_ powder and its mesoporous structure, in terms of crystallite size and pore connectivity, which better accommodated the volume expansion and in turn enabled the release of the deformation stress associated with the charging–discharging cycles.

Indeed, TiO_2_ electrode materials with large active surface areas allowed high electrochemical reaction rates per volume unit and high diffusion kinetics by reducing diffusion pathways for electronic and ionic transport, which could increase the battery capacity and cycling speed. Furthermore, as the crystallite size decreased and the pore volume increased, the ability of the mesoporous TiO_2_ powder to release the strain was stronger, which promoted good cycling stability. These results could be extended to other anode materials and cathode materials in Li-ion batteries.

Other questions remain unanswered in this work: which pore connectivity best mitigates the variation in electrode volume during the lithium insertion and de-insertion processes? In addition, from which crystallite size is their breakdown no longer possible?

## Figures and Tables

**Table 1:** Crystallite size of TiO_2_ powders prepared at various temperatures.

Temperature (°C)	Crystallite Size (nm)
170	17.9
190	17.7
210	15.7
230	14

**Table 2:** BET-specific surface area, mean pore diameter, and pore volume of the TiO_2_ powders.

Powder	BET-Specific Surface Area (m^2^/g)	Average Pore Diameter (nm)	Pore Volume (cm^3^/g)
TiO_2_-170 °C	65	4.0	0.07
TiO_2_-190 °C	75	4.0	0.11
TiO_2_-210 °C	77	4.5	0.12
TiO_2_-230 °C	103	4.5	0.26

**Table 3:** Crystallite size of TiO_2_ powders prepared at the different synthesis temperatures.

TiO_2_-24 h Crystallite Size (nm)	170 °C	190 °C	210 °C	230 °C
After synthesis	17.9	17.7	15.7	14
After 10th cycle	14.7	11.6	11.5	9.6

## Data Availability

The data presented in this study are available on request from the corresponding author.

## References

[1] Qu J., Ding J., Yuan N. (2015). The synthesis of four morphologies of TiO_2_ through temperature control and their electrochemical performance. Int. J. Elecrochem. Sci..

[2] Lu J., Chen Z., Pan F., Cui Y., Amine K. (2018). High-Performance anode materials for rechargeable lithium-ion batteries. Electrochem. Ener. Rev..

[3] Zhao Z., Tian J., Sang Y., Cabot A., Liu H. (2015). Structure, synthesis, and applications of TiO_2_ nanobelts. Adv. Mater..

[4] Xiang C., Li M., Zhi M., Manivannan A., Wu N. (2012). Reduced graphene oxide/titanium dioxide composites for supercapacitor electrodes: Shape and coupling effects. J. Mater. Chem..

[5] Yang Z., Zhang J., Kintner-Meyer M.C., Lu X., Choi D., Lemmon J.P., Liu J. (2011). Electrochemical energy storage for green grid. Chem. Rev..

[6] Armand M., Tarascon J.M. (2008). Building better batteries. Nature.

[7] Whittingham M.S. (2014). Ultimate limits to intercalation reactions for lithium batteries. Chem. Rev..

[8] Xu K. (2004). Nonaqueous liquid electrolytes for lithium-based rechargeable batteries. Chem. Rev..

[9] Xu K. (2014). Electrolytes and interphases in Li-ion batteries and beyond. Chem. Rev..

[10] Bruce P.G., Scrosati B., Tarascon J.-M. (2008). Nanomaterials for rechargeable lithium batteries. Angew. Chem. Int. Ed..

[11] Cabana J., Monconduit L., Larcher D., Palacín M.R. (2010). Beyond intercalation-based Li-ion batteries: The state of the art and challenges of electrode materials reacting through conversion reactions. Adv. Mater..

[12] Li H., Yang G., Miao X., Hong A. (2010). Efficient microwave hydrothermal synthesis of nanocrystalline orthorhombic LiMnO_2_ cathodes for lithium batteries. Electrochem. Acta.

[13] (2015). Thiophene derivatives as novel functional additives for high-voltage LiCoO_2_ operations in lithium-ion batteries. Electrochem. Acta.

[14] Muto M., Tatsumi K., Kojima Y., Oka H., Kondo H., Horibuchi K., Ukyo Y.Y. (2012). Effect of Mg-doping on the degradation of LiNiO_2_ based cathode materials by combined spectroscopic methods. J. Power Sources.

[15] Yin X., Huang K., Liu S., Wang H., Wang H. (2010). Preparation and characterization of Na-doped LiFePO_4_/C composites as cathode materials for lithium-ion batteries. J. Power Sources.

[16] Doi T., Yatomi S., Kida T., Okada S., Yamaki J.I. (2009). Liquid phase synthesis of uniformly nanosized LiMnPO_4_ particles and their electrochemical properties for lithium-ion batteries. Cryst. Growth Des..

[17] Han T.H., Lee W.J., Lee D.H., Kim J.E., Choi E.-Y., Kim S.O. (2010). Peptide/graphene hybrid assembly into core/shell nanowires. Adv. Mater..

[18] Aricò A.S., Bruce P., Scrosati B., Tarascon J.M., van Schalkwijk W. (2005). Nanostructured materials for advanced energy conversion and storage devices. Nat. Mater..

[19] Heidari E.-K., Kamyabi-Gol A., Heydarzadeh Sohi M., Ataie A. (2018). Electrode Materials for Lithium Ion Batteries: A Review. J. Ultrafine Grained Nanostruct. Mater..

[20] Cheng Q., Zhang Y. (2018). Multi-Channel Graphite for High-Rate Lithium Ion Battery. J. Electrochem. Soc..

[21] Huang Y., Yang H., Zhang Y., Zhang Y., Wu Y., Tian M., Chen P., Trout R., Ma Y., Wu T.-H. (2019). A safe and fast-charging lithium-ion battery anode using MXene supported Li_3_VO_4_. J. Mater. Chem. A.

[22] Hoffmann M.R., Martin S.T., Choi W., Bahnemann D.W. (1995). Environmental applications of semiconductor photocatalysis. Chem. Rev..

[23] Grätzel M. (2001). Photoelectrochemical cells. Nature.

[24] Wang C., Yin L., Zhang L., Gao R. (2010). Ti/TiO_2_ nanotube array/Ni composite electrodes for nonenzymatic amperometric glucose sensing. J. Phys. Chem. C.

[25] Armstrong G., Armstrong A.R., Bruce P.G., Reale P., Scrosati B. (2006). TiO_2_ (B) nanowires as an improved anode material for lithium-ion batteries containing LiFePO_4_ or LiNi_0.5_Mn_1.5_O_4_ cathodes and a polymer electrolyte. Adv. Mater..

[26] Yang X., Teng D., Liu B., Yu Y., Yang X. (2011). Nanosized anatase titanium dioxide loaded porous carbon nanofiber webs as anode materials for lithium-ion batteries. Electrochem. Commun..

[27] Qiao H., Xiao L., Zhang L. (2008). Phosphatization: A promising approach to enhance the performance of mesoporous TiO_2_ anode for lithium-ion batteries. Electrochem. Commun..

[28] Qiao H., Luo Q., Wei Q., Cai Y., Huang F. (2012). Electrochemical properties of rutile TiO_2_ nanorods as anode material for lithium-ion batteries. Ionics.

[29] Panda S.K., Yoon Y., Jung H.S., Yoon W.-S., Shin H. (2012). Nanoscale size effect of titania (anatase) nanotubes with uniform wall thickness as high-performance anode for lithium-ion secondary battery. J. Power Source.

[30] Ren Y., Liu Z., Pourpoint F., Armstrong A.R., Grey C.P., Bruce P.G. (2012). Nanoparticulate TiO_2_ (B): An anode for lithium-ion batteries. Angew. Chem. Int. Ed..

[31] Wang Y., Chen T., Mu Q. (2011). Electrochemical performance of W-doped anatase TiO_2_ nanoparticles as an electrode material for lithium-ion batteries. J. Mater. Chem..

[32] Ali Z., Cha S.N., Sohn J.I., Shakir I., Yan C., Kim J.M., Kang D.J. (2012). Design and evaluation of novel Zn doped mesoporous TiO_2_ based anode material for advanced lithium-ion batteries. J. Mater. Chem..

[33] Jung H.-G., Yoon C.S., Prakash J., Sun Y.-K. (2009). Mesoporous anatase TiO_2_ with high surface area and controllable pore size by F^-^-ion doping: Applications for high-power Li-ion battery anode. J. Phys. Chem. C.

[34] Xiao P.F., Lai M.O., Lu L. (2012). Electrochemical properties of nanocrystalline TiO_2_ synthesized via mechanochemical reaction. Electrochim. Acta.

[35] Pal M., Garcia Serrano J., Santiago P., Pal U. (2007). Size-controlled synthesis of spherical TiO_2_ nanoparticles: Morphology, crystallization, and phase transition. J. Phys. Chem. C.

[36] Bing Z., Yuan Y., Wang Y., Fu Z.-W. (2006). Electrochemical characterization of a three dimensionally ordered macroporous anatase TiO_2_ electrode. Electrochem. Solid-State Lett..

[37] Fu L.J., Zhang T., Cao Q., Zhang H.P., Wu Y.P. (2007). Preparation and characterization of three-dimensionally ordered mesoporous titania microparticles as anode material for lithium-ion battery. Electrochem. Commun..

[38] Lin Z., Yue W., Huang D., Hu J., Zhang X., Yuan Z.Y., Yang X. (2012). Pore lengh control of mesoporous Co3O4 and its influence on the capacity of porous electrodes for lithium-ion batteries. RSC Adv..

[39] Klug H.P., Alexander L. (1974). X-ray Diffraction Procedures: For Polycrystalline and Amorphous Materials.

[40] Taleb A., Mesguich F., Hérissan A., Colbeau-Justin C., Xue Y., Dubot P. (2016). Optimized TiO_2_ nanoparticle packing for DSSC photovoltaic applications. Sol. Energy Mater. Sol. Cells.

[41] Mehraz S., Luo W., Swiatowska J., Bezzazi B., Taleb A. (2021). Hydrothermal Synthesis of TiO2 Aggregates and Their Application as Negative Electrodes for Lithium-Ion Batteries: The Conflicting Effects of Specific Surface and Pore Size. Materials.

[42] Kruk M., Jaroniec M. (2001). Gas adsorption characterization of ordered organic-inorganic nanocomposite materials. Chem. Mater..

[43] Yang P., Zhao D., Margolese D.I., Chmelka B.F., Stucky G.D. (1998). Generalized syntheses of large-pore mesoporous metal oxides with semicrystalline frameworks. Nature.

[44] Saravanan K., Ananthanarayanan K., Balaya P. (2010). Mesoporous TiO_2_ with high packing density for superior lithium storage. Energy Environ. Sci..

[45] Kavan L., Rathouský J., Grätzel M., Shklover V., Zukal A. (2000). Surfactant-templated TiO_2_ (anatase): Characteristic features of lithium insertion electrochemistry in organized nanostructures. J. Phys. Chem. B.

[46] Kavan L., Kratochvilová K., Grätzel M. (1995). Study of nanocrystalline TiO_2_ (anatase) electrode in the accumulation regime. J. Electroanal. Chem..

[47] Stashans A., Lunell S., Bergström R., Hagfeldt A., Lindquist S.E. (1996). Theoretical study of lithium intercalation in rutile and anatase. J. Phys. Rev. B.

[48] Oh S.W., Park S.H., Sun Y.K. (2006). Hydrothermal synthesis of nano-sized anatase TiO_2_ powders for lithium secondary anode materials. J. Power Source.

[49] Sudant G., Baudrin E., Larcher D., Tarascon J.-M. (2005). Electrochemical lithium reactivity with nanotextured anatase-type TiO_2_. J. Mater. Chem..

[50] Jiang C., Wei M., Qi Z., Kudo T., Honma I., Zhou H. (2007). Particle size dependence of the lithium storage capability and high-rate performance of nanocrystalline anatase TiO_2_ electrode. J. Power Source.

[51] Kang J.W., Kim D.H., Mathew V., Lim J.S., Gim J.H., Kim J. (2011). Particle size effect of anatase TiO_2_ nanocrystals for lithium-ion batteries. J. Electrochem. Soc..

[52] Lee K.H., Song S.W. (2011). One-step hydrothermal synthesis of mesoporous anatase TiO_2_ microsphere and interfacial control for enhanced lithium storage performance. ACS Appl. Mater. Interfaces.

[53] Zhao M., Zhao D.L., Han X.Y., Yang H.X., Duan Y.J., Tian X.M. (2018). Ge nanoparticles embedded in spherical ordered mesoporous carbon as anode material for high performance lithium ion batteries. Electrochim. Acta.

